# The Role of Metal Ions in Biology, Biochemistry and Medicine

**DOI:** 10.3390/ma14030549

**Published:** 2021-01-24

**Authors:** Michael Moustakas

**Affiliations:** Department of Botany, Aristotle University of Thessaloniki, GR-54124 Thessaloniki, Greece; moustak@bio.auth.gr

Metal ions are fundamental elements for the maintenance of the lifespan of plants, animals and humans. Their substantial role in biological systems was recognized a long time ago. They are essential for the maintenance of life and their absence can cause growth disorders, severe malfunction, carcinogenesis or death. They are protagonists as macro or microelements in several structural and functional roles, participating in many biochemical reactions, and arise in several forms. They participate in intra and inter cellular communications, in maintaining electrical charges and osmotic pressure, in photosynthesis and electron transfer processes, in the maintenance of pairing, stacking and the stability of nucleotide bases, and also in the regulation of DNA transcription. They contribute to the proper functioning of nerve cells, muscle cells, the brain and the heart, the transport of oxygen and in many other biological processes up to the point that we cannot even imagine a life without metals.

In the present work, the 10 papers published in this Special Issue are summarized, providing a picture of metal ions uses in Biology, Biochemistry and Medicine, but also pointing out the toxicity impacts on plants, animals, humans, and the environment.

Metals (Cobalt (Co), Nickel (Ni) and Chromium (Cr)) are used in prosthesis manufacturing because of their high mechanic stability and good biological compatibility, but their corrosion products have been shown to elicit biological responses [1]. Monocytes and macrophages are the first barrier of the innate immune system, which interact with abrasion and corrosion products, leading to the release of proinflammatory mediators and reactive oxygen species (ROS) [1]. The inflammation-relevant changes in monocytes and macrophages after exposure to several concentrations of metal salts (CoCl_2_, NiCl_2_, CrCl_3_ × 6H_2_O) were studied by analyzing viability, gene expression, protein release and ROS production [1]. It was proved that monocytes and macrophages react very sensitively to corrosion products and that high concentrations of bivalent ions lead to cell death, while lower concentrations trigger the release of inflammatory mediators, mainly in macrophages [1].

Metal particles, as well as their corrosion products, have been shown to elicit a biological response in the aseptic loosening of endoprosthetic implants [2]. Owing to different metal alloy components, the response may vary depending on the nature of the released corrosion product [2]. Human osteoblasts were incubated with different metal salts (CoCl_2_, NiCl_2_ and CrCl_3_ × 6H_2_O) in order to compare the biological effects of different ions released from metal alloys [2]. The results demonstrated the pro-osteolytic capacity of metal ions in osteoblasts, while the metal ions examined intervene much earlier in inflammatory processes compared to CoCr_28_Mo_6_ particles [2].

Titanium (Ti), being one of the most abundant elements in the earth’s crust with many examples of bioactive properties, is widely used in the cosmetic industry. Titanium dioxide (TiO_2_) nanoparticles (NPs) are often incorporated in sunscreens as physical sun blockers absorbing ultraviolet (UV) radiation [3]. By examining the reactivity of TiO_2_ NPs in the presence and absence of UV, Sharma et al. [3] reassessed in a review article what their seepage into bodies of water can cause to the environment and aquatic life, and examined TiO_2_ NPs’ effects on human skin and health in general, and especially on the human body and the bloodstream.

Phenolic compounds play a significant role in plant tolerance to toxic metals, together with the prevention and reduction of biotic and abiotic oxidative stress [4]. Chlorogenic acid (5-caffeoylquinic acid, 5-CQA) is a phenolic compound considered to play an essential role in defense against biotic and abiotic stresses and is accumulated in plant tissues exposed to different stress factors that result in increased ROS production [4]. Zinc (Zn) cations and Zn complexes have anti-/pro-oxidant and antimicrobial activities [4]. The antimicrobial activity of the Zn(II) complex of 5-CQA and 5-CQA against *Escherichia coli*, *Pseudomonas aeruginosa*, *Bacillus subtilis*, *Staphylococcus aureus*, *Salmonella enteritidis* and *Candida albicans* was tested by Kalinowska et al. [4]. The pro-oxidant activity of Zn(II) 5-CQA was higher than the ligand and increased with the rise in the compound concentration [4]. The type of Zn(II) coordination by the chlorogenate ligand strongly affected the antioxidant activity of the complex [4].

The impact of Zn oxide nanoparticles (ZnO NPs) on photosystem II (PSII) photochemistry and ROS production was evaluated in the seagrass *Cymodocea nodosa* exposed to 5 and 10 mg L^−1^ ZnO NPs [5]. A disturbance of PSII functionality was observed 4 h after exposure to 10 mg L^−1^ ZnO NPs, but later (24 h) a stimulatory effect on PSII photochemistry was noticed and described as a hormetic response [5]. A hormetic response suggests that a basal stress level is needed for adaptive responses, which in the case of the seagrass *Cymodocea nodosa* exposed to ZnO NPs was found to be 10 mg L^−1^ ZnO NPs, with 24 h exposure time to be required for the induction of this adaptive response mechanism [5].

Both Zn and copper (Cu) are essential elements for plant growth, and an adequate supply of both is suggested to improve crop productivity. Sperdouli et al. [6] exposed young and mature *Arabidopsis thaliana* leaves to CuZn nanoparticles (NPs) in order to evaluate their effect on PSII function. PSII function in young *A. thaliana* leaves was detected to be negatively influenced by the foliar spray of CuZn NPs, while a beneficial effect on PSII function in mature leaves was observed. The explanation of the differential response of young and mature *Arabidopsis* leaves was suggested to be due to the nutrient remobilization that occurs in mature-senescing leaves, which results in nutrient deficiencies [6]. The use of CuZn NPs as foliar spray fertilizers to improve nutrient crop deficiencies is proposed, and it is concluded that leaf age must be taken into consideration in order to compare leaves of the same developmental stage [6].

Aluminium (Al), the most abundant metal in the earth’s crust, is not considered to be essential for plant life, but it can be toxic to plant growth in acid soils (pH < 5.5) at the ionic form of Al^3+^ species, limiting crop production through negatively affecting root growth, nutrient uptake and other metabolic processes, especially photosynthesis [7]. Moustaka et al. [7] evaluated the responses of photosystem II (PSII) and photosystem I (PSI) to Al^3+^ phytotoxicity in the durum wheat (*Triticum turgidum* L. cv. ‘Appulo E’) and the triticale (X Triticosecale Witmark cv. ‘Dada’) and concluded that PSII was more affected than PSI by Al^3+^ phytotoxicity. Thus, PSII is documented for another time to be the most sensitive component of the photosynthetic apparatus, playing key roles in photosynthetic responses to environmental perturbations.

Cadmium (Cd), a non-essential heavy metal to plants, can occur in soil at high concentrations, thus becoming toxic to all organisms. However, some plant species that can take up heavy metals and translocate them into the above-ground parts, in a manner that is not detrimental, are called hyperaccumulators, and they can be used for phytoremediation. *Noccaea caerulescens* can accumulate Zn–Cd–Ni at enormously high concentrations in its aboveground tissues, and has been suggested as a model species for examining metal tolerance and hyperaccumulation [8]. Bayçu et al. [8] provided new data on the mechanism of *N. caerulescens* acclimation to Cd exposure by elucidating the process of PSII acclimation. The authors concluded that acclimation to 120 μM Cd exposure, possibly achieved through a reduced plastoquinone (PQ) pool signaling, mediated stomatal closure, probably by the generation of mesophyll chloroplastic hydrogen peroxide (H_2_O_2_). Stomatal closure decreased Cd supply at the spatially affected leaf area, while it was suggested that H_2_O_2_ signaling activated the Cd detoxification mechanism through its vacuolar sequestration. As a result, a lower ROS production as singlet oxygen (^1^O_2_) was detected [8]. It was concluded that the response of *N. caerulescens* to Cd exposure fits the ‘Threshold for Tolerance Model’, with a lag time of 4 days and a threshold concentration of 40 μM Cd to be required for the induction of the acclimation mechanism [8].

Chlorophyll fluorescence analysis has been commonly used as an extremely sensitive marker of photosynthetic efficiency [9]. Nevertheless, photosynthesis is not uniform at the leaf area, denoting conventional chlorophyll fluorescence measurements as non-characteristic of the physiological status of the entire leaf. This disadvantage overcomes chlorophyll fluorescence imaging analysis, which permits the detection of spatiotemporal heterogeneity at the total leaf surface [9]. By combining the chlorophyll fluorescence imaging analysis (CF-IA) and laser ablation inductively coupled plasma mass spectrometry (LA-ICP-MS) methods, the impact of Cd accumulation on the photosynthetic efficiency of *Salvia sclarea* was examined [9]. The spatial heterogeneity of a decreased effective quantum yield of electron transport (Φ*_PSΙΙ_*), which was observed after exposure to Cd, was linked to the spatial pattern of high Cd accumulation [9]. However, the increased photoprotective heat dissipation (NPQ) in the whole leaf under Cd exposure was sufficient enough to retain the same fraction of open reaction centers (*q*_P_) as control leaves, also resulting in a decreased quantum yield of non-regulated energy loss (Φ*_NO_*), even more than that of control leaves, demonstrating the tolerance of *S. sclarea* to Cd exposure [9]. The results revealed the advantages of combining CF-IA and LA-ICP-MS to monitor heavy metal effects on plants and to elucidate plant tolerance mechanisms [9].

Mercury (Hg) is a toxic metal frequently used in the illegal extraction of gold and silver, consequently resulting in environmental poisoning [10]. *Lysinibacillus sphaericus* strains characterized by scanning electron microscopy (SEM) coupled with energy dispersive spectroscopy (EDS-SEM) were assessed for Hg removal ability [10]. Sorption was evaluated in live and dead bacterial biomass by free and immobilized cells assays. EDS-SEM analysis showed that the bacteria strains used could adsorb Hg as particles of nanometric scale, removing over 95% of Hg, suggesting that *L. sphaericus* could be used as a novel biological method of mercury removal from polluted wastewater [10].
